# The development of compulsive coping behavior depends on dorsolateral striatum dopamine-dependent mechanisms

**DOI:** 10.1038/s41380-023-02256-z

**Published:** 2023-09-28

**Authors:** Lucia Marti-Prats, Chiara Giuliano, Ana Domi, Mickaël Puaud, Yolanda Peña-Oliver, Maxime Fouyssac, Colin McKenzie, Barry J. Everitt, David Belin

**Affiliations:** 1 Behavioural and Clinical Neuroscience Institute and Department of Psychology, University of Cambridge, Downing Street, Cambridge, CB2 3EB UK; 2 Present Address: Astra Zeneca, R&D Biopharmaceuticals, Fleming Building (B623), Babraham Research Park, Babraham, Cambridgeshire, CB22 3AT UK; 3 Present Address: Department of Psychiatry and Neurochemistry, Institute of Neuroscience and Physiology, Sahlgrenska Academy University of Gothenburg, Box 410, Gothenburg, 405 30 Sweden; 4 Present Address: Research and Enterprise Services, University of Sussex, Brighton, UK

**Keywords:** Addiction, Neuroscience

## Abstract

Humans greatly differ in how they cope with stress, a natural behavior learnt through negative reinforcement. Some individuals engage in displacement activities, others in exercise or comfort eating, and others still in alcohol use. Across species, adjunctive behaviors, such as polydipsic drinking, are used as a form of displacement activity that reduces stress. Some individuals, in particular those that use alcohol to self-medicate, tend to lose control over such coping behaviors, which become excessive and compulsive. However, the psychological and neural mechanisms underlying this individual vulnerability have not been elucidated. Here we tested the hypothesis that the development of compulsive adjunctive behaviors stems from the functional engagement of the dorsolateral striatum (DLS) dopamine-dependent habit system after a prolonged history of adjunctive responding. We measured in longitudinal studies in male Sprague Dawley rats the sensitivity of early established vs compulsive polydipsic water or alcohol drinking to a bilateral infusion into the anterior DLS (aDLS) of the dopamine receptor antagonist α-flupentixol. While most rats acquired a polydipsic drinking response with water, others only did so with alcohol. Whether drinking water or alcohol, the acquisition of this coping response was insensitive to aDLS dopamine receptor blockade. In contrast, after prolonged experience, adjunctive drinking became dependent on aDLS dopamine at a time when it was compulsive in vulnerable individuals. These data suggest that habits may develop out of negative reinforcement and that the engagement of their underlying striatal system is necessary for the manifestation of compulsive adjunctive behaviors.

## Introduction

When facing the distress generated by a challenging, emotionally taxing, aversive situation, individuals greatly differ in the emotion regulation strategy they use, which is influenced by situational demands [1–6]. Emotion regulation and associated coping strategies [7], such as exercise, comfort eating, shopping, or displacement behavior [8, 9], all of which are driven by the negative reinforcing effects of stress reduction, have long been suggested to be a prerequisite for adaptive functioning, the promotion of resilience and well-being [10, 11].

Several species cope with stress using a form of displacement called adjunctive behavior [12, 13]. One such adjunctive anxiolytic response, schedule-induced polydipsia (SIP) [12–15], manifests itself as polydipsic water intake in the face of intermittent food delivery in food-restricted animals [16–18]. At the population level, non-regulatory polydipsic drinking develops over a week and remains stable for long periods of time during which it selectively decreases the levels of stress-related hormones that had been increased by the associated intermittent food delivery [14, 15, 19–21].

However, some humans [22–29] and individuals of other species [30] characterized, for instance by a high impulsivity trait [31], lose control over these coping strategies which become excessive and promote the development of compulsive disorders such as obsessive compulsive and substance use disorders.

During emotion regulation challenges under circumstances often perceived as overwhelming, such as were posed by the COVID-19 pandemic [32–35], some individuals resort to alternative means to cope with stress, such as drinking alcohol [36–40] or use of other drugs [41]. This in turn is associated with a greater vulnerability to develop compulsivity [40, 42–50]. Across species the individual tendency to rely on the anxiolytic effects of alcohol to cope with stress [42, 50, 51], including that induced by the SIP procedure [52], has been associated with an increased vulnerability to switch from controlled to compulsive alcohol use, a key feature of alcohol use disorder (AUD) [42, 49, 50, 52–54].

However, the psychological and neural basis of the individual vulnerability to lose control over coping behavior, whether involving alcohol use or not, and the ensuing development of compulsivity are unclear.

Increasing evidence suggests that the transition from controlled, goal-directed, behavior to compulsion, including the compulsive seeking and drinking of alcohol [55, 56] and the development of compulsive adjunctive polydipsic drinking, is dependent on a shift in the locus of control over behavior from the ventral to the dorsolateral striatum (DLS)-dependent habit system [57]. Thus, while the reinforcing properties of alcohol, mediated by the mesolimbic dopamine (DA), system support recreational alcohol use [58, 59], it is the engagement of anterior DLS (aDLS) DA dependent alcohol seeking habits that promotes the transition to compulsive alcohol seeking and drinking [55, 56, 59]. Similarly, the development of adjunctive polydipsic water drinking behavior, but not its compulsive manifestation, is dependent on the mesolimbic system [60, 61]. In contrast, well-established excessive adjunctive water drinking (hyperdipsia) in vulnerable individuals has been associated with an increased in spine density in DLS medium spiny neurons [62].

Here we tested the hypothesis that the development of compulsive coping behavior, manifested as excessive polydipsic drinking, whether of alcohol or water, is associated with the functional engagement of, and an increased reliance on, the DLS DA-dependent habit system.

To test this hypothesis, we assessed the sensitivity of the polydipsic water or alcohol drinking response of each individual in large cohorts of outbred rats to bilateral aDLS infusions of the DA receptor antagonist α-flupentixol [52, 56, 63] over the course of the acquisition of each coping behavior and the subsequent transition to compulsivity.

## Methods and materials

### Subjects

One hundred and forty-one male Sprague Dawley rats (Charles River, UK), weighing 300–350 g at the start of the experiments, were used in this study as described in detail in the *SOM*. All experimental protocols were conducted under the project license 70/8072 held by David Belin in accordance with the regulatory requirement of the UK Animals (Scientific Procedures) Act 1986, amendment regulations 2012, following ethical review by the University of Cambridge Animal Welfare and Ethical Review Body (AWERB).

### Experimental procedures

**Fig. 1:**
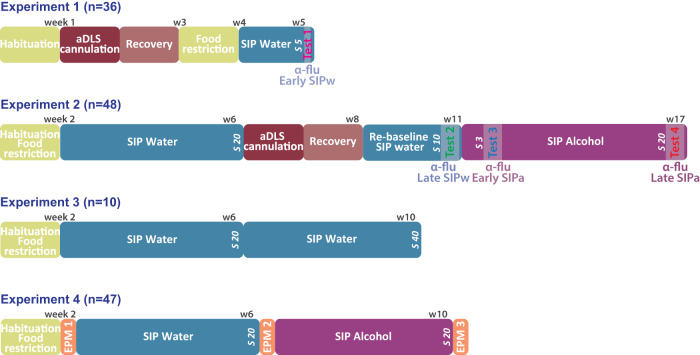
Timeline of the experiments. Timeline of the four experiments carried out on independent cohorts of male Sprague Dawley rats. *Experiment 1***:** Following a week of habituation to the animal facility, thirty-six rats received bilateral cannulation of their anterior dorsolateral striatum (aDLS). A week later, rats were food restricted to 85% of their theoretical free-feeding weight and trained in a schedule-induced polydipsia (SIP) procedure with water (SIPw). The reliance of the acquisition of adjunctive water drinking on aDLS dopaminergic mechanisms was assessed after 5 SIPw sessions as the sensitivity of drinking behavior to bilateral infusion of the dopamine (DA) receptor antagonist α-flupentixol (α-flu, 0, 6, 12 μg/side, between-subject design) into the aDLS (*Test 1, α-flu Early SIPw*). *Experiment 2*: Following a week of habituation to the animal facility, forty-eight rats were food restricted for a week before being trained in the SIPw procedure for 20 sessions, e.g., until the establishment of hyperdipsia in vulnerable individuals. Then, rats were implanted with bilateral cannulae targeting the aDLS and, after at least a week, they were re-baselined under SIPw for 10 sessions. Then, the reliance of well-established, compulsive, adjunctive water drinking behavior on aDLS DA was assessed as the sensitivity of water drinking behavior to bilateral infusion of α-flupentixol (0, 6, 12 μg/side, within-subject design) into the aDLS (*Test 2, α-flu Late SIPw*). Subsequently, water was replaced by 10% alcohol (SIPa) and the reliance of early and well-established adjunctive alcohol drinking on aDLS DA was measured after 3 *(Test 3, α-flu Early SIPa*) or 20 (*Test 4, α-flu Late SIPa*) SIPa sessions, respectively. *Experiment 3*: In order to test the stability of water intake levels once adjunctive water drinking has been established, ten rats were food restricted after a week of habituation to the colony, and then trained in the SIP procedure for 40 sessions, a period of training similar to that used in experiment 2, but with water as the only available solution throughout. *Experiment 4*: In order to establish the anxiolytic properties of polydipsic drinking under SIP at the population level but also, specifically in individuals that relied on alcohol to engage in a coping strategy, forty-seven rats were habituated to the animal facility and food restricted for a week before their anxiety levels were assessed as the percentage of time spent in the open arms of an elevated plus maze (EPM) prior to (naïve, EPM 1), and immediately following a session after at least 20 days of training under SIPw (EPM 2) or SIPa (EPM 3).W: week; s: session.

The series of experiments are schematically summarized in Fig. 1.

Experiments 1 and 2 aimed to establish the reliance of coping behavior on a circuit that involves aDLS DA-dependent mechanisms (referred to subsequently as aDLS DA), an established assay that reveals the engagement of habitual control over behavior reinforced by addictive drugs, including alcohol [55, 56, 63]. Thus inactivation of, or DA receptor blockade in, the aDLS does not influence early performance of alcohol, cocaine or heroin seeking behavior [63–67], which instead is mediated by the posterior dorsomedial striatum (pDMS)-dependent goal-directed system [63, 64, 67]. In contrast, the later emerging habitual responding for these drugs becomes sensitive to the same aDLS manipulations that had no effect on early performance [55, 56, 63, 64, 67–69]. These data reveal a double dissociation in the control over behavior by the aDLS- and pDMS-dependent corticostriatal networks mediating habitual and goal-directed control over behavior, respectively [63, 67, 70].

As detailed in Fig. 1 and the *SOM,* the first experiment aimed to determine the involvement of aDLS DA in the acquisition of a coping adjunctive water drinking response (SIPw) (*Test 1, Effect of α-flu on Early SIPw*).

The second experiment aimed to test the reliance on aDLS DA of well-established adjunctive water drinking (*Test 2, Effect of α-flu on Late SIPw*) vs that of early (*Test 3, Effect of α-flu on Early SIPa*) and well-established adjunctive alcohol drinking (SIPa) (*Test 4, Effect of α-flu on Late SIPa*), as previously described [52].

The third experiment aimed to test the stability of polydipsic water intake levels over a period of training in the SIP procedure similar to that of rats in experiment 2.

The fourth experiment aimed to test the anxiolytic nature of polydipsic alcohol drinking at the population level but also, specifically in individuals that relied on alcohol to engage in a coping strategy.

### Schedule-induced polydipsia (SIP)

SIP training was carried out as previously described [31, 71, 72] and detailed in the *SOM*.

The SIP procedure was based on a fixed-time 60 s (FT-60 s) schedule of food delivery, previously shown to induce polydipsic drinking with robust and persistent individual differences in the tendency to develop excessive, compulsive manifestations of this adjunctive behavior [31, 52, 72–74]. Individuals having an average water consumption over the last 3 days of training in the upper and lower quartiles of the population were considered as high (HD) and low drinkers (LD), respectively, as previously described [31, 52, 72]. HD rats are therefore individuals who acquire a coping response with water (e.g., water copers) and tend, for some of them, to lose control over it, thereby expressing hyperdipsia. LD rats, on the other hand do not readily acquire a coping response with water. However, fifty to sixty percent of them drink alcohol to do so, these are alcohol copers [52].

Twenty-four hours after the last SIPw session, water was replaced by 10% alcohol and rats were trained for twenty 60 min SIPa sessions. The total amount of alcohol consumed (mL) was calculated daily as the difference between the weights of the bottle before and after the session.

### Drugs

The alcohol and DA receptor antagonist α-flupentixol solutions were prepared as previously described [52, 65] and detailed in the *SOM*.

### Surgery: aDLS cannulations

Rats received bilateral aDLS implantation of canulae under stereotaxic surgery either before behavioral training (Experiment 1), or after acquisition of SIPw (Experiment 2) under isoflurane anesthesia (O2: 2 L/min, 5% isoflurane for induction and 2% for maintenance), as previously described [56] and detailed in the *SOM*.

### Intra-striatal infusions

The influence of aDLS DA receptor blockade on adjunctive drinking behavior was tested at early and late stage of training for SIPw and SIPa, namely during the acquisition of SIPw (Experiment 1, SIPw session 5 onwards, *Test 1 Effect of α-flu on Early SIPw*) and SIPa (Experiment 2, SIPa session 3 onwards, *Test 3, Effect of α-flu on Early SIPa*) and well-established SIPw (Experiment 2, SIPw session 20 onwards, *Test 2, Effect of α-flu on Late SIPw*) and SIPa (SIPa session 20 onwards, *Test 4, Effect of α-flu on Late SIPa*).

As described in the *SOM*, each test session was preceded by intra-aDLS infusions (0.5 μl/side) of α-flupentixol (0, 6, 12 μg/side, made via 28-gauge steel injectors (Plastics One, Roanoke, VA, USA) over 90 s followed by a 60 s period to allow diffusion of the infused drug or vehicle before injectors were removed and obturators were replaced. Test sessions began 5 min later. The effect of aDLS DA receptor blockade on adjunctive drinking was tested on rats from experiment 1 (*Test 1*) in a between-subject design, and in rats from experiment 2 (*Tests 2, 3 and 4*) in a counter-balanced order following a Latin-Square design. Each infusion day was followed by two baseline sessions.

### Histology

As described in detail in the *SOM*, at the end of the experiment, rats were euthanized, perfused transcardially with 10% neutral buffered formalin and their brains were extracted before being cryoprotected and then processed into 60 μm coronal sections that were mounted and stained with Cresyl Violet. Cannulae placements in the aDLS were verified using a light microscope by an experimenter blind to the behavioral results.

### Elevated plus maze

Anxiety levels were assessed as the percentage of time spent in the open arms of an elevated plus maze (EPM) prior to (naïve), and immediately following a session after at least 20 days of training under SIPw or SIPa, as previously described [31] and detailed in the *SOM*.

### Data and statistical analyses

Data, presented as means ± SEM, individual data points or box plots [medians ± 25% (percentiles) and Min/Max as whiskers], were analyzed with STATISTICA-10 Software (Statsoft, Inc., Tulsa, OK, USA) or Statistical Package for Social Sciences (IBM SPSS, v 26, USA) as described in the *SOM*.

Behavioral data on the acquisition or maintenance of SIPw or SIPa were analyzed using repeated measures (RM) analyses of variance (ANOVA).

The effect of aDLS DA receptor blockade on adjunctive drinking was analyzed with one way- (*Test 1*) or RM-ANOVA (*Test 2, 3 and 4*).

Two-step K-mean cluster analysis [56, 71] was performed to identify groups of individuals whose reliance of adjunctive drinking on aDLS DA differed across tests. We additionally computed an index of differential reliance on aDLS DA of compulsive drinking of alcohol vs water to analyze whether the emerging reliance on aDLS control predicts the individual tendency to rely on alcohol to cope with stress and the ensuing development of compulsive alcohol drinking [52].

Rats were also ranked according to their fluid intake (mL) before receiving α-flupentixol infusions (i.e., after the last three session of SIPw or SIPa) to identify individuals that increased their intake when alcohol was introduced in the SIP procedure.

Changes in anxiety level over the course of exposure to SIP were computed as the relative change (%) in the percentage of time spent in the open arms of the maze from one test to the next. A one-tailed *t*-test was used to test the hypothesis that the introduction of alcohol resulted in a decrease in anxiety levels specifically in individuals that relied on alcohol to develop a coping response. Pearson’s correlations were used to investigate relationships between the magnitude of polydipsic fluid intake and the level of anxiety.

On confirmation of significant main effects, the effect sizes of which are reported as partial eta squared (η_p_^2^), differences among individual means were further analyzed using post-hoc tests or planned comparisons, as appropriate. Significance was set at α ≤ 0.05.

## Results

### Individual differences in the reliance on alcohol to engage in a coping strategy

**Fig. 2:**
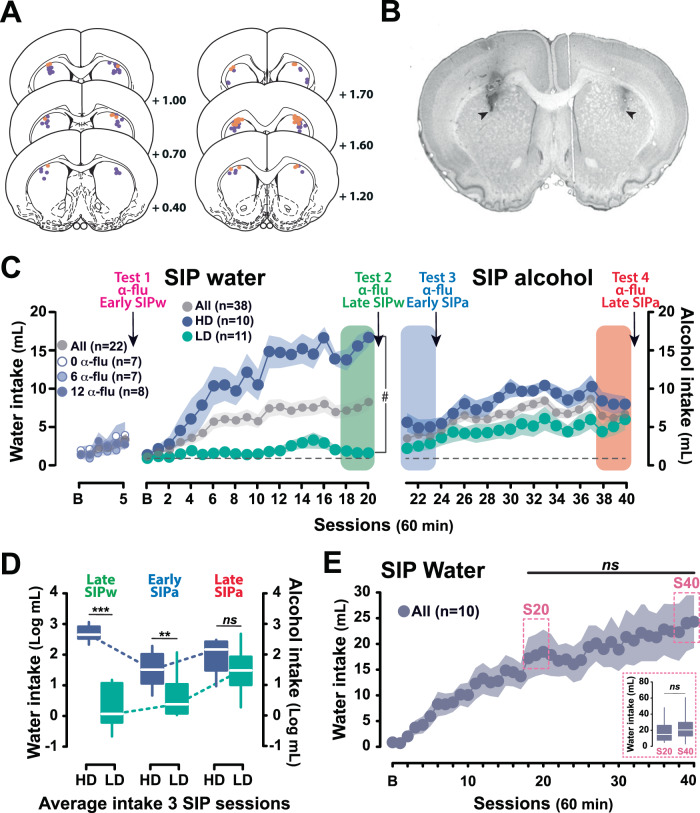
Individual differences in the tendency to develop compulsive adjunctive behaviors in rat. **A**, **B** Rats having completed experiments 1 and 2 with cannula tips (orange and violet for experiment 1 and 2, respectively) located in the anterior dorsolateral striatum (aDLS) according to the rat brain atlas (black arrows) [149] as assessed following staining with Cresyl Violet were included in the final analyses. **C**, **D** At the population level, and similarly across each independent group for experiment 1, food restricted rats exposed to intermittent food delivery progressively developed adjunctive polydipsic water drinking over five (Experiment 1, *n* = 22) and twenty (Experiment 2, *n* = 38) sessions. Experiment was designed to reveal marked individual differences in the tendency to engage in polydipsic water drinking that emerged early on in training. While at the population level individuals developed a polydipsic water drinking response, some individuals, developed an excessive, compulsive polydipsic behavior while others did not engage in a displacement strategy whatsoever. Thus, high drinker (HD) and low drinker rats (LD), selected respectively in the upper and lower quartiles of the population stratified on the average water consumption over the last 3 sessions of SIP with water (Late SIPw, green rectangle), greatly differed in their trajectory of polydipsic water drinking. HD rats developed a compulsive water drinking behavior that reached more than 16.65 ± 1.08 ml/h by the last session, or two times more than the whole population, whereas LD rats maintained throughout a water drinking behavior similar to that associated with their homeostatic need displayed at baseline (**B**). The introduction of alcohol resulted in significant changes in coping behavior. Thus, HD and LD rats still differed in their level of adjunctive drinking at the beginning of the SIP training with alcohol (Early SIPa, blue rectangle), mostly due to the fact that HD rats, who had developed an hyperdipsia with water persisted following the introduction of alcohol, albeit to a lower level. However, while HD rats maintained overall a steady level of polydipsic alcohol drinking over time, LD rats acquired a coping response with alcohol and eventually developed compulsive polydipsic alcohol drinking so that they no longer differed from HD rats by the end of training (Late SIPa, orange rectangle). **E **This increase in alcohol drinking shown by LD rats was not attributable to an increase in fluid intake over time since once adjunctive drinking was established in an independent cohort of rats over 20 sessions (Experiment 3, *n* = 10), polydipsic water drinking remained stable so that by session 40 (*S40* dashed-line rectangle, average of sessions 38–40) rats displayed a similar level of fluid intake as they did by the end of the first 20 session period (*S20* dashed-line rectangle, average of sessions 18–20). The reliance of early (Tests 1 and 3) and well-established (Tests 2 and 4) polydipsic water or alcohol drinking on aDLS DA was assessed as the sensitivity of drinking behavior to bilateral infusion of α-flupentixol (α-flu) at each time point identified by an arrow. ♯ *p* < 0.001 group × time interaction; *** *p* < 0.001, ** *p* < 0.01, HD different from LD rats; ns: no significant.

Sixty rats with cannula placements located in the aDLS were included in the final analysis (Fig. 2A, B).

Exposure to the SIP procedure resulted, at the population level, in a progressive development of adjunctive drinking behavior, expressed as an increase in water intake in response to the introduction of a FT- 60 s schedule of food delivery over 5 (for *Experiment 1*) or 20 sessions (for *Experiment 2*) [main effect of time: *F*_2.7,55.8_ = 5.46, *p* = 0.003, η_p_^2^ = 0.21 and *F*_4.4,162.4_ = 17.89, *p* < 0.001, η_p_^2^ = 0.33, respectively]. Polydipsic water intake began differing from baseline prandial water drinking from FT-60 s session 4 and 3 onwards, respectively (Fig. 2C).

As previously described [31, 52, 72, 75], marked individual differences in the propensity to lose control over adjunctive water drinking behavior emerged over 20 SIPw sessions [main effect of group: *F*_1,19_ = 52.73, *p* < 0.001, η_p_^2^ = 0.73; time: *F*_20,380_ = 16.39, *p* < 0.001, η_p_^2^ = 0.46 and group × time interaction: *F*_20,380_ = 11.74, *p* < 0.001, η_p_^2^ = 0.38] (Fig. 2C). Thus, HD rats (upper quartile of the population, *n* = 10) were prone readily to develop hyperdipsia, eventually drinking more than 15 mL/h (15.30 ± 1.21 mL over the last three sessions). This represents more than twice the intake of the population as a whole, and almost ten times more than that of LD rats (lower quartile of the population, *n* = 11) (1.70 ± 0.32 mL over the last three sessions) [main effect of group: *F*_1,19_ = 113.19, *p* < 0.001, η_p_^2^ = 0.86] (Fig. 2D, left panel). This differential level of polydipsic water intake observed between HD and LD rats after 20 daily SIP sessions remained stable over long periods of time, as previously shown [31].

However, the introduction of the opportunity to drink alcohol instead of water as a means to cope with the stress induced by the SIP procedure resulted in an asymmetrical change in the level of adjunctive drinking displayed by LD and HD rats [main effect of time: *F*_2,38_ = 9.86, *p* < 0.001, η_p_^2^ = 0.34; group: *F*_1,19_ = 39.81, *p* < 0.001, η_p_^2^ = 0.68 and time × group interaction: *F*_2,38_ = 22.95, *p* < 0.001, η_p_^2^ = 0.55] (Fig. 2D). Thus, despite an overall decrease in total fluid intake upon the introduction of alcohol, reflective of its anxiolytic properties [76], HD and LD rats initially maintained their then long established difference in the level of adjunctive drinking [HD (5.14 ± 0.84 mL/h) vs LD (2.54 ± 0.69 mL/h, *p* = 0.006] (Fig. 2D, middle panel). However, over the 20 sessions of SIPa some LD rats (63.6%) more than doubled their intake of alcohol, eventually reaching 5.14 ± 1.12 mL per hour [LD, Early SIPa vs Late SIPa, *p* < 0.001] (Fig. 2D). These alcohol coper LD rats that relied on alcohol to develop a coping response contributed greatly to the overall increase in alcohol intake shown at the group level [main effect of time *F*_9.2,340_ = 12.49, *p* < 0.001, η^2^ = 0.25] (Fig. 2C). Alcohol coper LD rats became as compulsive as HD rats [Late SIPa, HD vs LD, *p* = 0.09], which only increased their alcohol intake by ~50% over the same period [HD, Early SIPa vs Late SIPa, *p* < 0.02] (Fig. 2D, right panel).

**Fig. 3:**
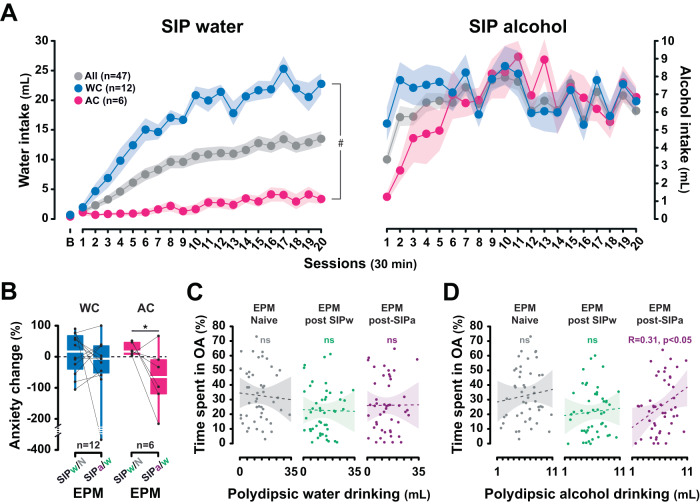
Alcohol drinking under SIP is anxiolytic, especially in individuals that rely on alcohol to engage in a coping strategy. **A** At the population level, individuals exposed to a SIP procedure developed a polydipsic drinking coping response with water that they maintained over time. However, some individuals (High drinker rats  or Water coper rats, WC, upper quartile of the population) developed an excessive compulsive polydipsic drinking behavior while others (Low drinker rats, LD) did not acquire this adjunctive behavior. Similarly to experiment 2, while WC rats maintained overall a steady level of polydipsic drinking over time when alcohol was introduced in place of water, some LD rats acquired a coping response with alcohol (alcohol coper rats, AC) and eventually developed compulsive polydipsic alcohol drinking so that they eventually no longer differed from WC rats after 20 SIPa sessions. **B** Trajectories in anxiety levels (assessed as the percentage of time in the open arms of an elevated plus maze in a naïve state or immediately following a SIP session with water (SIPw) or alcohol (SIPa) after at least 20 daily sessions each) revealed that the polydipsic alcohol drinking displayed by the LD rats that relied on alcohol to engage in a coping strategy (AC) resulted in a decrease in their anxiety levels, as compared to those measured after a SIPw session. **C**, **D** Furthermore, at the population level, a negative correlation emerged between the level of polydipsic drinking and anxiety following the introduction of alcohol, which revealed the anxiolytic nature of polydipsic alcohol drinking. *p < 0.05, AC, SIPw/N vs SIPa/w; #*p* <0.001, time x group x liquid interaction; ns: not significant. B: baseline session; EPM: elevated plus maze; N: naïve state; SIPw/N: change in anxiety levels after being trained in the SIPw vs the naïve state; SIPa/w: change in anxiety levels after being trained in SIPa vs SIPw.

The sudden increase in fluid consumption shown by alcohol coper LD rats upon the introduction of alcohol cannot be accounted for by a simple increase in fluid intake over time since once established, polydipsic water intake remained stable over protracted periods of time (e.g., at least 40 days) [main effect of session: *F*_40,360_ = 9.49, *p* < 0.001, η_p_^2^ = 0.51; from session 15, between sessions all *p* values > 0.05] (Fig. 2E). Thus, rats exposed to the SIP procedure displayed similar level of polydipsic water intake over sessions 38–40 as over sessions 18–20 [*F*_1,9_ = 2.80, *p* = 0.13, η^2^ = 0.24] (Fig. 2E, insert). Instead, as shown in an independent replication on a cohort of 47 individuals [main time × group × liquid interaction: *F*_19,304_ = 10.33, *p* < 0.001, η_p_^2^ = 0.39] (Fig. 3A), the emergence of polydipsic alcohol drinking in the LD rats that relied on alcohol to engage in a coping strategy (alcohol copers) resulted in a decrease in their anxiety level, as assessed on an EPM [One tailed *t-*test: *t* = −2.25, *p* < 0.05] (Fig. 3B). The anxiolytic properties of the development of an adjunctive response using alcohol drinking were further supported at the population level by the emergence of a negative correlation between the level of polydipsic drinking and anxiety measured immediately after a SIP session following the introduction of alcohol (Fig. 3C, D) [*R* = 0.31, *p* < 0.05].

These results together replicate both previous evidence of individual differences in the tendency to lose control over stress reducing polydipsic water drinking [52, 77, 78] and our original demonstration of individual differences in the reliance on alcohol to cope with stress, the latter being a gateway for the development of excessive and compulsive alcohol drinking [52].

We then sought to identify the neural locus of control of these coping behaviors that appear initially to be goal-directed, the goal of the response (drinking) being the alleviation of stress [14, 21, 79], but that eventually become excessive and compulsive [75, 80, 81]. This transition is hypothesized here to reflect the development of maladaptive negative-reinforcement driven habits. Thus, we assessed the reliance of adjunctive fluid drinking on aDLS DA, a signature of habitual control over behavior, including alcohol-related responding [64], and the compulsion to seek and drink alcohol [55, 56].

### Hyperdipsia is associated with the functional engagement of the aDLS DA-dependent habit system

**Fig. 4:**
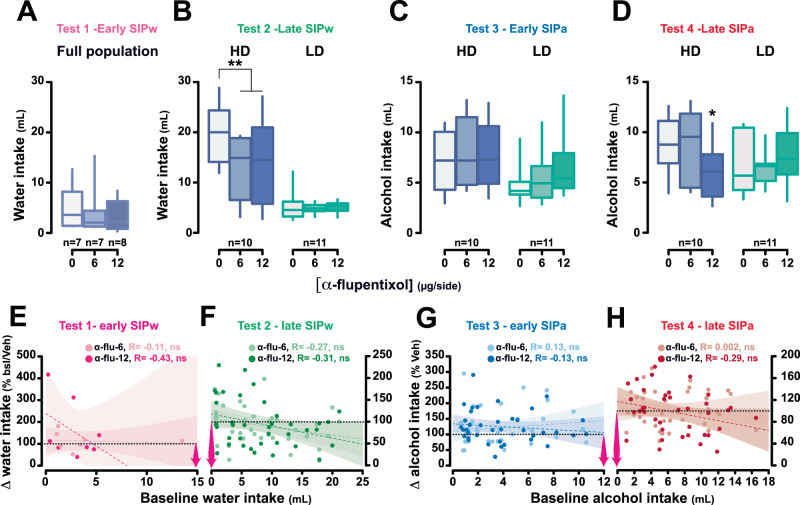
Excessive, but not early, adjunctive drinking is dependent on anterior dorsolateral striatum dopamine-dependent mechanisms. Intra-anterior dorsolateral striatum (aDLS) infusion of the dopamine receptor antagonist α-flupentixol (0, 6, 12 μg/side) had no effect on recently acquired polydipsic water drinking (Test 1, **A**) or on drinking shown by low drinker (LD) rats even after 20 sessions (Test 2, **B**). In contrast, similar intra aDLS infusions of α-flupentixol (0, 6, 12 μg/side) decreased excessive water intake in high drinkers (HD) rats (Test 2, **B**). When water was replaced by alcohol, intra aDLS dopamine receptor blockage no longer influenced polydipsic drinking in HD rats while it remained ineffective in LD rats (Test 3, **C**). In marked contrast, the sensitivity shown by HD rats to intra aDLS dopamine receptor blockade when engaged in excessive polydipsic water intake re-emerged when their polydipsic alcohol intake became well established (Test 4, **D**). **E–H** The functional engagement of the aDLS DA-dependent habit system when polydipsic water or alcohol drinking becomes compulsive, namely at tests 2 and 4, is not due to a floor effect inherent to relatively lower levels of non-prandial drinking at tests 1 and 2 since no correlations were observed between the magnitude of the decrease in fluid intake following intra aDLS infusion of either dose of α-flupentixol and the baseline level of water (**E**, **F**) or alcohol (**G**, **H**) adjunctive drinking. ** *p* < 0.01, * *p* < 0.05 different from vehicle (0 μg/side). SIPw: SIP with water; SIPa: SIP with alcohol; α-flu: α-flupentixol. ∆water intake (%bsl/veh): difference in water intake following aDLS DA receptor blockage and baseline (bsl, for test 1) or intra aDLS infusion of vehicle (for test 2). ∆alcohol intake (%bsl): difference in alcohol intake following aDLS DA receptor blockage vs intra aDLS infusion of vehicle (tests 3 and 4). In **E**, **F** pink arrows indicate the spread of the reliance on the aDLS DA-dependent system.

As predicted, after a short-term exposure to SIPw, prior to the development of excessive drinking behavior in vulnerable individuals, aDLS DA receptor blockade with α-flupentixol (0, 6, 12 μg/0.5 μl/side; between-subject design) had no effect on water intake [main effect of treatment: *F*_2,19_ = 2.11, *p* = 0.13, η_p_^2^ = 0.10] (*Test 1, Early SIPw*, Fig. 4A). However, after extended exposure to SIPw, at a time when vulnerable individuals had developed compulsive adjunctive drinking, the same aDLS DA receptor blockade (0, 6, 12 μg/0.5 μl/side α-flupentixol; within-subject counter-balanced design) now resulted in a marked decrease in adjunctive water intake in these HD rats [HD vs LD: main effect of treatment: *F*_2,38_ = 4.16, *p* = 0.023, η_p_^2^ = 0.18; group: *F*_1,19_ = 44.85, *p* < 0.001, η_p_^2^ = 0.70 and treatment × group interaction: *F*_2,38_ = 2.96, *p* = 0.06, η_p_^2^ = 0.13].

Follow-up analyses confirmed that aDLS DA receptor blockade did not influence drinking in LD rats [main effect of treatment: *F*_2,20_ = 0.31, *p* = 0.74, η_p_^2^ = 0.03], while it greatly reduced excessive adjunctive drinking in HD rats [main effect of treatment: *F*_2,18_ = 3.55, *p* = 0.049, η_p_^2^ = 0.28; 0 vs 6 and 12 µg/side: *p* = 0.007] (*Test 2, Late SIPw,* Fig. 4B).

In marked contrast, in the same rats, the introduction of alcohol as a means to cope with stress resulted in a disengagement, albeit transient (see below), of aDLS DA control over adjunctive drinking. Thus, intra aDLS infusions of the DA receptor antagonist α-flupentixol no longer decreased fluid intake in HD rats after 3 days of SIPa [*HD vs LD*: main effect of group: *F*_1,19_ = 4.95, *p* = 0.038, η_p_^2^ = 0.21, treatment: *F*_2,38_ = 1.44, *p* = 0.25, η_p_^2^ = 0.07 and treatment × group interaction: *F*_2,38_ = 0.90, *p* = 0.42, η_p_^2^ = 0.04] (*Test 3, Early SIPa*, Fig. 4C). These results thereby demonstrate that learning to drink alcohol to cope with stress, even in individuals that had established a compulsive adjunctive drinking behavior with water, is associated with a disengagement of habitual control over behavior.

However, following 20 daily sessions of SIPa when adjunctive alcohol drinking had escalated and become excessive, it became, once again, reliant on aDLS DA, as shown by the emergence of a sensitivity of alcohol drinking to aDLS DA receptor blockade in HD rats [*HD vs LD*: main effect of group: *F*_1,19_ = 1.17, *p* = 0.29, η_p_^2^ = 0.06 and treatment x group interaction: *F*_2,38_ = 4.76, *p* = 0.014, η_p_^2^ = 0.20; HD rats, 0 vs 12 μg/side: *p* = 0.044] (*Test 4, Late SIPa*, Fig. 4D). The functional engagement of the aDLS DA-dependent habit system selectively when polydipsic water or alcohol drinking becomes compulsive cannot be explained by a floor effect in early water or alcohol drinking stages since the magnitude of the decrease in fluid intake following intra aDLS infusion of either dose of α-flupentixol was not related to the baseline level of drinking (Fig. 4E–H).

### Differential trajectories of the functional engagement of the habit system predict individual differences in the reliance on drinking alcohol to engage a coping strategy

Building on previous evidence in a positive reinforcement setting that the reliance of habitual alcohol seeking on aDLS DA predicts the severity of the ensuing compulsive behavior [56], here we further investigated whether a similar relationship was observed between the reliance of adjunctive responses, maintained through negative reinforcement, on aDLS DA and the vulnerability to develop excessive, compulsive adjunctive drinking.

We capitalized on the within-subject design of experiment 2 to determine, for each individual, the differential sensitivity of excessive adjunctive drinking of water (*Test 2*) vs alcohol (*Test 4*) to intra aDLS infusions of α-flupentixol (mean % decrease of intake following DA receptor blockade relative to baseline, i.e., following vehicle infusions).

**Fig. 5:**
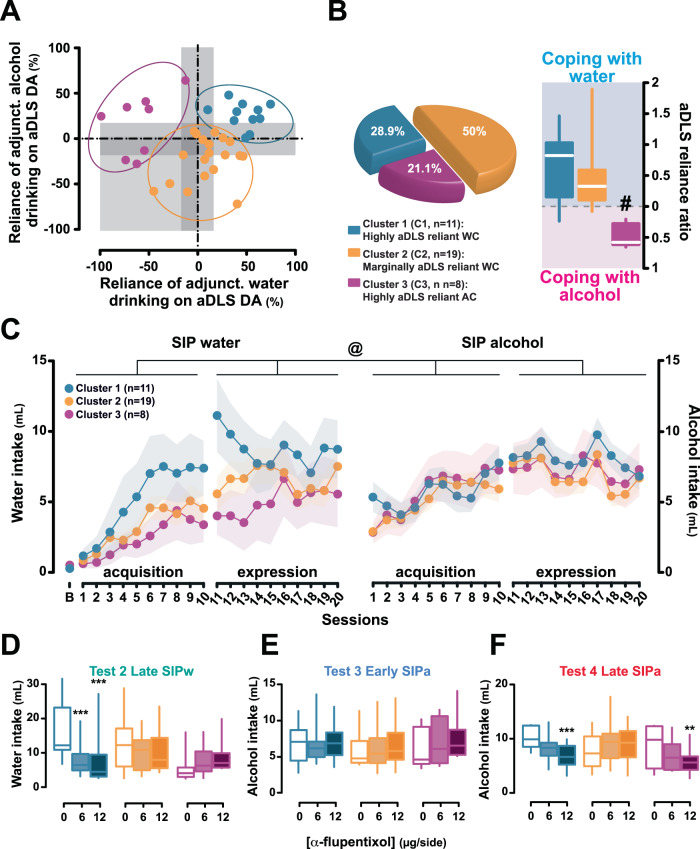
The individual tendency to develop anterior dorsolateral striatum dopamine-dependent-coping response is associated with the vulnerability to develop compulsive adjunctive behavior. **A**, **B** Marked individual differences in the reliance of compulsive drinking on anterior dorsolateral striatum (aDLS) dopamine (DA) were revealed when normalizing the influence of aDLS DA receptor blockade on polydipsic water (Late SIP water) or alcohol drinking (Late SIP alcohol) to the baseline levels of drinking following vehicle infusions (α-flupentixol 0 μg/side). A cluster analysis identified three subpopulations of rats, one (Cluster 1) comprised individuals whose polydipsic behavior, irrespective of the fluid drank, was heavily reliant on aDLS DA. These highly aDLS reliant water copers (WC) represented 28.9% of the overall population and consisted predominantly (82%) of high drinkers (HD) and intermediate individuals. A second cluster (Cluster 2) comprised individuals whose polydipsic water or alcohol drinking behavior was overall marginally sensitive to aDLS DA receptor blockade. These marginally aDLS reliant WC represented 50% of the population and consisted predominantly (84%) of low drinkers (LD) and intermediate rats. The third cluster (Cluster 3) comprised individuals whose polydipsic alcohol drinking was much more reliant on aDLS DA than their polydipsic water drinking behavior. These highly aDLS reliant alcohol coper (AC) rats represented 21% of the population and consisted predominantly (87.5%) of LD and intermediate rats. A systematic analysis of the respective reliance of polydipsic water vs alcohol drinking behavior on aDLS DA confirmed that only highly aDLS reliant AC rats displayed a selective increased in the engagement of aDLS DA-dependent control over behavior when they used alcohol as a mean to cope with stress. **C** Retrospective analysis of the acquisition and expression of polydipsic water and/or alcohol drinking of these three clusters confirmed that Cluster 1 WC rats developed hyperdipsia with water while Cluster 3 AC rats did so with alcohol. **D–F** Marked differences were observed in the time course of the engagement of the aDLS DA-dependent habit system in mediating compulsive coping behavior in the individuals of these three groups that map perfectly those observed on HD and LD rats (Fig. 3), in that rats from Cluster 1 showed sensitivity to aDLS DA receptor blockade when SIPw (Test 2 Late SIPw) and SIPa (Test 4 Late SIPa) were well-established whereas rats from Cluster 3 showed such sensitivity only when SIPa was well-established, neither showing reliance on aDLS DA during the acquisition of SIPa. Rats from the heterogenous Cluster 2 never showed a decrease in adjunctive drinking following aDLS that reached statistical significance throughout the training history. *** *p* ≤ 0.001, ** *p* ≤ 0.01, compared with the vehicle treatment (0 μg/side); # *p* < 0.001 compared with cluster 1 and 2; @: main cluster × liquid × period interaction, *p* < 0.001.

A K-mean cluster analysis [55, 71] revealed three sub-populations that differed in the reliance of their excessive water or alcohol adjunctive drinking on aDLS DA (Fig. 5A). A first cluster (Cluster 1, 28.9% of the total population) comprised individuals that had all developed an aDLS DA-dependent adjunctive water drinking behavior before they switched to alcohol drinking, which also became reliant on aDLS DA.

These aDLS reliant water coper rats, which comprised 82% of HD and intermediate individuals, differed from rats of Cluster 2 (50% of the population; which instead comprised 84% of intermediate and LD rats) whose water drinking was marginally reliant on aDLS DA and who did not engage their habit system when subsequently exposed to alcohol.

In contrast, individuals in Cluster 3 (21.1% of the population, which comprised 87.5% of LD and intermediate rats) were the only ones to develop an adjunctive response reliant on aDLS DA when they used alcohol, as confirmed by an analysis of the ratio of sensitivity of adjunctive fluid intake to aDLS DA receptor blockade between SIPa and SIPw [main effect of cluster: *F*_2,35_ = 13.33, *p* < 0.001, η_p_^2^ = 0.43; Cluster 3 vs 1 and 2, *p* < 0.001, respectively) (Fig. 5B, right panel).

Retrospective investigation of the individuals comprising each cluster revealed that most of those belonging to Cluster 1 were water coper HD rats whereas the majority of individuals in Cluster 3 were alcohol coper LD rats that had instead developed hyperdipsia after the introduction of alcohol. This was further supported by the analysis of the acquisition and expression of polydipsic water and/or alcohol drinking of these three clusters (Fig. 5C): aDLS DA reliant water copers and aDLS DA reliant alcohol copers (cluster 1 and 3, respectively) had different coping styles accompanied by different trajectories in the development of their hyperdipsia [main cluster × fluid (water vs alcohol) × period (acquisition vs expression) interaction: *F*_18,315_ = 2.64, *p* < 0.001, η_p_^2^ = 0.13]. Cluster 1 water coper rats developed hyperdipsia with water while cluster 3 alcohol coper rats did so with alcohol, increasing their polydipsic alcohol intake by 60% over the 3 weeks of SIPa, eventually to reach the level of intake shown by water coper rats.

The time course of the engagement of aDLS DA in the control over adjunctive behavior of Cluster 3 alcohol coper rats was therefore very different to that shown by Cluster 1 water coper rats (Fig. 5D–F) [treatment x time x cluster interaction: *F*_5,87.3_ = 4.48, *p* = 0.001, η_p_^2^ = 0.20; treatment × time interaction: *F*_2.5,87.3_ = 4.48, *p* = 0.005, η_p_^2^ = 0.12], with profound differences in their sensitivity to aDLS DA receptor antagonism at Late SIPw (*Test 2*, Fig. 5D) [main effect of treatment: *F*_1.5,52.2_ = 5.01, *p* = 0.017, η_p_^2^ = 0.12; treatment × cluster interaction: *F*_3,52.2_ = 6.16, *p* = 0.001, η_p_^2^ = 0.26] and at Late SIPa (*Test 4*, Fig. 5F) [main effect of treatment: *F*_1.7,60.1_ = 6.49, *p* = 0.004, η_p_^2^ = 0.16; treatment × cluster interaction: *F*_3.4,60.1_ = 7.55, *p* < 0.001, η_p_^2^ = 0.30], but not Early SIPa (*Test 3*, Fig. 5E) [main effect of treatment: *F*_2,70_ = 1.22, *p* = 0.30, η_p_^2^ = 0.03; treatment × cluster interaction: *F*_4,70_ = 0.21, *p* = 0.93, η_p_^2^ = 0.01].

The differences at Late SIPw were driven exclusively by Cluster 1 water coper individuals [0 vs 6 μg/side: *p* < 0.001, 0 vs 12 μg/side: *p* < 0.001] (Fig. 5D), whereas the effects at Late SIPa were driven by Cluster 1 water coper and Cluster 3 alcohol coper individuals [*Cluster 1*, 0 vs 12 μg/side: *p* = 0.001; *Cluster 3*, 0 vs 12 μg/side: *p* = 0.010] (Fig. 5F).

Together these data demonstrate that the development of compulsive adjunctive drinking depends on the engagement of aDLS DA-dependent control over behavior that eventually occurred in 81.6% of the individuals in this study.

## Discussion

At the population level, individuals in the present study developed an adaptive coping response under a SIP procedure [75], expressed as non-regulatory adjunctive polydipsic drinking behavior. While exposure to intermittent food delivery in food restricted individuals activates the hypothalamic-pituitary-adrenal (HPA) axis [13] and increases anxiety [31], the development of polydipsic water drinking, which was shown here to remain stable over up to 42 days, has long been shown to reduce the activity of the HPA axis [52, 77, 78].

However, within 2 weeks some individuals progressively lost control over the polydipsic drinking response and developed hyperdipsia, a compulsive coping strategy [74, 75, 80–82]. In these HD rats, no changes in anxiety levels were seen following SIPw, revealing that in these individuals, even though it has become compulsive, coping behavior remains acutely anxiolytic [77] and mitigates the otherwise anxiety-inducing effects of the procedure [31]. This observation lends additional support to the construct validity of hyperdipsia as a model of compulsive behavior, since in compulsive disorders compulsions remain acutely anxiolytic in the context of an overall stress surfeit [83].

In contrast, some individuals did not acquire a coping response with water, but only did so when they had access to alcohol. These alcohol coper LD rats learnt to drink alcohol under the anxiogenic effects of intermittent food delivery, but had not done so by engaging in polydipsic water drinking. Moreover, they increased their daily intake of alcohol more rapidly than any other group, and eventually developed compulsive coping behavior. Engaging in polydipsic alcohol drinking, which was shown here to decrease anxiety in alcohol copers, has previously been shown to decrease plasma corticosterone concentration [84] at the population level. It has also been shown to be unrelated to ethanol preference or a tendency to drink more alcohol outside the SIP context [52, 84] and also cannot be solely accounted for by any direct effect of alcohol on the HPA axis [84]. Since it is the stress-reducing properties of the adjunctive response and not the fluid ingested that maintains polydipsic drinking under negative reinforcement, exposure to SIPa does not influence the reinforcing properties of alcohol in a different context [85].

The results of the present study replicate previously reported individual variability in the tendency both to engage in polydipsic drinking [52] and to develop compulsive adjunctive behaviors [31, 52, 72–75, 80, 81, 86], and more recent evidence that some individual rats that do not develop a coping response with water, do so readily when drinking alcohol [52].

In addition, the results of the present study provide causal evidence of a progressive engagement of aDLS DA in the control over adjunctive behavior, especially when it becomes compulsive in vulnerable individuals. While the acquisition of polydipsic drinking of water or alcohol was not dependent on aDLS DA, its excessive and compulsive manifestation in vulnerable individuals was selectively decreased by bilateral aDLS DA receptor blockade at a time it has previously been shown no longer to be sensitive to dopaminergic manipulations of the mesolimbic system [61].

In alcohol copers, this engagement of aDLS DA habit system [87, 88] in the control over polydipsic alcohol drinking was specific to a facilitated transition to compulsion by acquiring alcohol use as a self-medication strategy. It could not be attributed to the emergence of habitual control over behavior by protracted training [89] because:

(i) the individual differences observed in the tendency to develop excessive polydipsic drinking after 20 sessions of SIPw did not evolve further across an additional period of 20 sessions when animals continued to be given access to water instead of alcohol, see [31],
(ii) and some individuals that had not engaged aDLS DA when drinking under SIPw also did not do so when subsequently given access to alcohol (Fig. 5).

Finally, the emerging reliance on aDLS DA of alcohol drinking as a coping behavior cannot be explained by a difference in the level of fluid intake: while the level of compulsive fluid intake shown by HD rats at the end of the 20 sessions of SIPa was much lower than that shown at the end of the 20 sessions of SIPw, in both cases their excessive adjunctive behavior was equally sensitive to aDLS DA receptor blockade. In addition, as shown in Fig. 4, the reliance on aDLS control is not predicated on the level of fluid intake.

Importantly, although they decreased the volume of fluid they drank when alcohol was introduced, HD rats still ingested 5–8 ml/h of alcohol, which is more than double the volume drunk by the same strain of rats (1–3 ml/h) following at least 12 sessions of intermittent access to 10% alcohol in a two-bottle choice procedure, data from [52], a time when rats had already escalated their alcohol intake. These observations reveal the excessive nature of their alcohol intake under SIP [90], which has previously been shown to result in blood alcohol levels higher than 0.6 g.l^−1^ [52].

These results are consistent with a body of evidence suggesting marked differences in the psychological and neural mechanisms underlying the acquisition of adjunctive coping responses in males and its subsequent compulsive manifestation in vulnerable individuals. However, women differ from men in coping strategies and in the impact of stress on their alcohol use or misuse [91, 92], and female rodents develop higher levels of SIP for water [93, 94] or alcohol [95] than males. Thus, further research is warranted to investigate whether the psychological and neural mechanisms identified here that result in the development of compulsive adjunctive behaviors in males may be even more readily engaged in females.

Because adjunctive behaviors do not lead to the delivery of food or help to meet homeostatic need, for review see [79], they have long been considered not to be instrumental. However, since the outcome of coping strategies is not the procurement of an external outcome, but a change in an interoceptive state, namely anxiolysis [96], adjunctive behavior, manifested here as polydipsic drinking, which superficially may appear to be consummatory, is in fact an instrumental response [79, 97, 98]. However, this instrumental response relies, like comfort eating, on the association between the ingestive behavior and stress reduction. Hence, the polydipsic drinking is elicited and maintained by the negative reinforcing properties of stress/anxiety reduction (Fig. 3 and [77]) and its associated interoceptive mechanisms [99].

This view is supported by evidence that the engagement in polydipsic drinking during the first week of exposure to SIP depends on the interoceptive cortex, namely the anterior insula [31], and results in a decrease in the activation of the HPA axis provoked by intermittent food delivery [14, 15, 19–21, 77]. Furthermore, at the same early stage of training, prevention of drinking in the SIP context results in an increase in corticosterone levels [13].

Early polydipsic water intake has features of goal-directedness in that it is sensitive to outcome devaluation (anti-anxiolysis), since there is a decrease in responding following administration of anxiogenic drugs such as CRF [100] or amphetamine [61, 101]. In contrast, following prolonged exposure to SIP, the same acute amphetamine challenge no longer results in a decrease in drinking [101]. These observations suggest that polydipsic drinking is initially a response tied to the motivational value of its outcome, namely anxiolysis, but that it eventually becomes habitual [102].

Development of habitual control over polydipsic drinking is consistent with a progressive shift in the striatal locus of control over behavior under SIP from a network involving the ventral and dorsomedial striatum to one involving the DLS [55, 56, 63, 70, 103]. Selective 6-hydroxydopamine lesions of the mesolimbic system prevent the acquisition of SIP [104–106], which is otherwise associated with a ramping of extracellular levels of DA in the nucleus accumbens core across sessions [107]. In contrast, DA transporter deficient mice and rats, which have elevated levels of DA, display an impaired acquisition of polydipsic drinking [108, 109], which is also decreased in wild type animals by the acute administration of psychostimulants such as cocaine and D-amphetamine directly into the nucleus accumbens [30, 110]. Together these results show that the acquisition of adjunctive drinking behavior depends on DA signalling in the mesolimbic system, but that it is impaired by an increase in tonic extracellular levels of DA.

In marked contrast, when this coping behavior is well established and becomes excessive in vulnerable individuals, it is no longer impaired by similar causal manipulations of the mesolimbic DA system [61]. Furthermore, repeated amphetamine exposure, which facilitates habitual responding under positive reinforcement [111], exacerbates polydipsic drinking [112] and renders it independent of food delivery [112, 113], suggesting the development of stimulus-response control over behavior [114] when it has become compulsive in vulnerable individuals. These observations are in line with the evidence that polydipsic drinking during early SIPw training is not accompanied by neuronal activation in the DLS [115], whereas its compulsive manifestation after extended exposure is associated with increased spine density in this region [62].

The apparent shift from a network involving the ventral striatum to one involving the DLS in the locus of control over adjunctive behaviors when they become compulsive [116] agrees with previous evidence that the development of compulsive alcohol seeking in vulnerable individuals depends on the prior functional engagement of aDLS DA [56]. This lends further support to the hypothesis that the development of compulsion stems from a loss of control over aDLS-dependent habits [117–120].

While stress shifts the balance toward aDLS-dependent habits from ventral striatal-dependent, goal-directed, behavior [121–123], it is unlikely that individual differences in the tendency to develop compulsive polydipsic water drinking observed here and elsewhere [31, 72–74] are due to a differential sensitivity to stress. Individuals that did not acquire a coping response with water did so readily when they had access to alcohol instead. This suggests that the lack of development of an adjunctive behavior under SIPw in these individuals was not due to their lack of sensitivity to the stress engendered by the SIP procedure. Instead, these individuals needed alcohol in order to develop this coping response.

In contrast, the tendency of water copers to develop a coping response with water and the subsequent loss of control over drinking in vulnerable individuals may reflect an interplay between a heightened sensitivity to negative urgency [124] and an inherent propensity generally to rely on the aDLS habit system. Hence, on the one hand, the development of compulsive SIP depends on both insula-dependent interoceptive mechanisms, the noradrenaline stress system and their interaction with pre-existing impulsivity [31, 72]. Whereas on the other hand, it is also predicted by a pre-existing tendency to use the habit system across a wide array of tasks, from response learning in spatial navigation [125] to resistance to devaluation in instrumental reinforcement and perseverative responses in reversal learning task [115, 126]. Overall, these results support the view that the inflexibility and increased responding under operant schedules that promote habitual behavior shown by high water drinker (HD) rats [74, 127] arises from an interaction between an inherent tendency to rely on the habit system and its recruitment by negative reinforcement during the development of a coping response.

Using the same SIP procedure, we have previously shown that alcohol enables the development of adjunctive responses in a specific subpopulation of rats otherwise unable to cope with stress by drinking water. In this subpopulation of LD rats, the phenotype of which was also identified independently in experiment 4 of the present study, it is the acquisition of alcohol use as a self-medication strategy, and not the overall level of intoxication (alcohol coper rats did not differ from water copers in their overall level of alcohol intake or the blood alcohol levels they reached) that determined their greater vulnerability to develop compulsive, quinine resistant, alcohol drinking [52]. This subpopulation of alcohol coper rats is very similar to that identified as Cluster 3 in the present study which only engaged aDLS DA when alcohol was introduced in the SIP procedure.

The engagement of an aDLS DA-dependent circuit in these compulsive alcohol drinkers is consistent with previous evidence of the recruitment of this circuitry following chronic exposure to alcohol and other addictive drugs in humans [59, 128], non-human primates [129, 130] and rodents [56, 63–66, 103, 131–133]. This may in turn be attributable to the adaptations that alcohol exposure causes in the aDLS, including its disinhibition by the dampening of GABAergic transmission onto its principal medium spiny projection neurons [134, 135].

Together, these observations support the view that the engagement of habitual control over negative-reinforcement driven behavior, such as the acquisition of alcohol drinking as a self-medication strategy, is an important determinant of the vulnerability to develop compulsive drinking [50, 54, 70, 118, 136].

It will be important to determine whether such negative reinforcement-driven, stress-bound maladaptive coping habits develop similarly when individuals learn to take drugs to alleviate negative states of withdrawal [137], and the extent to which they facilitate relapse under states of heightened stress [138, 139], as suggested by Marlatt’s taxonomy [140–142].

It remains also to be established why some individuals can only develop a coping response with alcohol but not water. One hypothesis is that the anxiolytic properties of alcohol (Fig. 3B–D) [143, 144] facilitate the acquisition of the adjunctive response in these individuals [145, 146]. This is also supported by the marked decrease in total fluid intake shown by water copers after the introduction of alcohol, which is quantitatively similar to that seen following administration of benzodiazepines [147].

The introduction of alcohol in these water coper rats resulted in a transient disengagement of the aDLS DA-dependent habit system. This reveals that the same behavioral expression of a coping response can be mediated by either the goal-directed or the habit system, as is the case for instrumental responses for positive reinforcers [65]. But perhaps more importantly it suggests that incentive learning can influence even a well-established, negative reinforcement-based habitual coping response [102, 148] when alcohol is adjunctively drunk, as has also been shown in rats self-administering heroin under a state of withdrawal [137].

Together these observations provide evidence for a role of negative reinforcement-based habits in the development of compulsive coping behaviors.

### Supplementary information

Supplementary materials
