# Supplementary material for: The development of compulsive coping behavior depends on dorsolateral striatum dopamine-dependent mechanisms
Source: Mol Psychiatry. 2023 Sep 28;28(11):4666–78. doi: 10.1038/s41380-023-02256-z (PMC10914627; doi:10.1038/s41380-023-02256-z)
Supplement: Supplementary file 1 — Supplementary materials [file 41380_2023_2256_MOESM1_ESM.docx]

# The development of compulsive coping behavior depends on dorsolateral striatum dopamine-dependent mechanisms

# Authors:

Lucia Marti-Prats^1 *^, Chiara Giuliano^1,2 *^, Ana Domi^1,3^, Mickaël Puaud^1^, Yolanda Peña-Oliver^1,4^, Maxime Fouyssac^1^, Colin McKenzie^1^, Barry J. Everitt^1^, David Belin^1^

^1^ Behavioural and Clinical Neuroscience Institute and Department of Psychology, University of Cambridge, Downing Street, Cambridge CB2 3EB, UK.

^2^ Current address: Astra Zeneca, R&D Biopharmaceuticals, Fleming Building (B623), Babraham Research Park, Babraham, Cambridgeshire CB22 3AT, UK.

^3^ Current address: Department of Psychiatry and Neurochemistry, Institute of Neuroscience and Physiology, Sahlgrenska Academy University of Gothenburg, Box 410, Gothenburg 405 30, Sweden.

^4^ Current Address: Research and Enterprise Services, University of Sussex, Brighton, UK.

* co-first authors

Corresponding author:

Dr David Belin

Department of Psychology,
University of Cambridge
Downing St.
Cambridge CB2 3EB, UK
Tel. +44 (0)1223333588

Fax. +44 (0)1223 333564
email: bdb26@cam.ac.uk

# Supplementary online materials

# Methods and Materials

## Subjects

One hundred and forty-one male Sprague Dawley rats (Charles River, UK), from four different cohorts, weighing 300-350g (~9-11 weeks old) at the start of the experiments, were used in this study. Rats were single-housed under a 12-hour reversed light/dark cycle (lights off at 7:00 AM) and food restricted to gradually reach 85% of their theoretical free-feeding body weight before the start of the behavioral training. Water was always available *ad libitum*. Experiments were performed 6-7 days/week between 8 am-5 pm.

All experimental protocols were conducted under the project license 70/8072 held by David Belin in accordance with the regulatory requirement of the UK Animals (Scientific Procedures) Act 1986, amendment regulations 2012, following ethical review by the University of Cambridge Animal Welfare and Ethical Review Body (AWERB).

## Experimental procedures

The series of experiments conducted in this study are schematically summarised in **Figure 1**.

The first experiment aimed to determine the involvement of aDLS DA-dependent mechanisms (referred to subsequently as aDLS DA) in the acquisition of a coping adjunctive water drinking response. Thus, after a week of habituation to the vivarium, thirty-six rats received bilateral cannulations of the aDLS and, following recovery, were food restricted to progressively reach 85% of their theoretical free-feeding weight. Rats were then trained in the SIP procedure with water (SIPw). Following one habituation and one baseline session (see SIP section below), rats were exposed to five 60-min SIPw sessions before the sensitivity of their early adjunctive water drinking to aDLS DA receptor blockade was measured (**Test 1, Effect of α-flu on Early SIPw**). Fourteen rats were excluded from the experiment because of loss of their guide cannula or cannula misplacements, so that twenty-two rats were included in the final analysis.

The second experiment aimed to test the reliance on aDLS DA of well-established adjunctive water drinking vs that of early and well-established adjunctive alcohol drinking. Thus, following a week of habituation to the vivarium, forty-eight rats were food restricted to progressively reach 85% of their theoretical free-feeding weight before starting behavioral training. Following one habituation and one baseline session (see SIP section below), rats were exposed to twenty 60-min SIPw sessions, e.g., until the establishment of excessive water intake in vulnerable individuals (1-4). Rats were subsequently implanted bilaterally with intra aDLS cannulae and, following recovery, they were re-baselined under SIPw for ten sessions before the reliance of their well-established adjunctive water drinking on aDLS DA was measured (**Test 2, Effect of α-flu on Late SIPw**). Then, water was replaced by 10% alcohol and rats were trained to maintain their adjunctive behavior now using alcohol (SIP with alcohol, SIPa), or to acquire the coping response for those that rely on alcohol to cope with distress, as previously described (4). The reliance of early and well-established adjunctive alcohol drinking on aDLS DA was measured after three (**Test 3, Effect of α-flu on Early SIPa**) or twenty daily sessions (**Test 4, Effect of α-flu on Late SIPa**), respectively.

Because ten individuals lost their cannulae or had misplaced cannulae as revealed after post-mortem histological assessment, thirty-eight rats were included in the final analysis.

The third experiment aimed to test the stability of polydipsic water intake levels over a period of training in the SIP procedure similar to that of rats in experiment 2. Thus, after a week of habituation to the vivarium, ten rats were food restricted to progressively reach 85% of their theoretical free-feeding weight before starting behavioral training. Following one habituation and one baseline session (see SIP section below), rats were exposed to forty 60-min SIPw sessions.

The fourth experiment aimed to test the anxiolytic nature of polydipsic alcohol drinking at the population level but also, specifically in individuals that relied on alcohol to engage in a coping strategy. Thus, after a week of habituation to the vivarium, the anxiety levels of each individual of an independent cohort of forty-seven male Sprague Dawley rats were assessed three times on an elevated plus maze (EPM): prior to (naïve), and immediately after exposure to 20 sessions of SIPw as well as immediately after 20 sessions of SIPa.

## Schedule-induced polydipsia (SIP)

### Apparatus

SIP training was carried out as previously described (1, 3, 5) in twelve operant chambers located within ventilated sound-attenuating cubicles (Med associates, St. Albans, USA) and made of aluminium and transparent acrylic plastic with a stainless-steel grid floor (24 cm x 25.4 cm x 26.7 cm). Chambers were equipped with a house light (3-W), a food tray (magazine) installed at the centre of the front wall, and a bottle from which a stainless-steel sipper tube delivered water or alcohol into a receptacle placed in a magazine on the wall opposite the food magazine. Water or alcohol were freely available throughout the 60-min (experiments 1, 2, 3) or 30-min sessions (experiment 4).

### Behavioral training

SIP training consisted of the following stages:

### Habituation / baseline water intake

During the habituation session, rats were given access to water and sixty food pellets (45 mg, TestDiet, USA) that had been placed in the magazine. The volume of water consumed by each rat over this session was measured in order to determine the amount of water each individual drank to meet their homeostatic needs while eating sixty 45 mg pellets. Rats were then tested in one magazine training session during which sixty food pellets were delivered under a variable interval 60-second schedule (VI-60 s) over 60 minutes (experiments 1, 2 and 3) or VI-30s over 30 minutes (experiment 4). This magazine training provided the baseline level of homeostatic water intake over one hour.

### SIP with water (SIPw)

The SIP procedure was based on a fixed-time 60-second (FT-60 s) or FT-30 s schedule of food delivery for the 60-min or 30-min sessions respectively, previously shown to induce adjunctive drinking behavior with robust and persistent individual differences in the tendency to develop excessive, compulsive adjunctive drinking behavior (1, 3, 4, 6, 7).

Twenty-four hours after the baseline session, rats underwent five (Experiment 1), twenty (Experiment 2 and 4) or forty (Experiment 3) of these 60- or 30-min FT-60 s or FT-30 s SIPw training sessions. 300 mL bottles filled with fresh water were weighed and inserted into the operant box immediately prior to the initiation of each session. House lights were switched on at the beginning and switched off at the end of each session. The total amount of water consumed (mL) was calculated daily as the difference between the weights of the bottle before and after the session.

Individuals whose average water consumption over the last three days of training was in the upper and lower quartiles of the population were considered as high (HD) and low drinkers (LD), respectively, as previously described (1, 3, 4).

### SIP alcohol (SIPa)

Twenty-four hours after the last SIPw session, water was replaced by 10% alcohol and rats were trained for twenty 60-min (experiment 2) or 30-min (Experiment 4) SIPa sessions. The total amount of alcohol consumed (mL) was calculated daily as the difference between the weights of the bottle before and after the session.

## Drugs

The alcohol solution was prepared by mixing 99.8% ethanol (Sigma-Aldrich, UK) with tap water to obtain 10% alcohol (4, 5).

The DA receptor antagonist α-flupentixol (Sigma-Aldrich, UK) was dissolved in double-distilled water as previously described (8). Drug doses (6 or 12 μg/side), reported in the salt form were selected to be in the range of those previously shown effectively to decrease aDLS-dependent cocaine, heroin or alcohol seeking behavior (8-11).

## Surgery: aDLS cannulations

Rats underwent stereotaxic surgery either before behavioral training (Experiment 1), or after acquisition of SIPw (Experiment 2) under isoflurane anaesthesia (O2: 2L/min, 5% isoflurane for induction and 2 % for maintenance), as previously described (10). Guide cannulae (22-gauge, Plastics One, Roanoke, VA, USA) were bilaterally implanted 2 mm above the aDLS (anterior/posterior (AP) +1.2, mediolateral (ML) ±3, dorsal/ventral (DV) -3; AP and ML coordinates measured from bregma, DV coordinates from the skull, incisor bar at −3.3 mm), as previously described (10, 12). Cannulae were held in place using dental acrylic cement anchored to four stainless steel screws tapped into the frontal and parietal bones of the skull. Obturators (Plastics One, Roanoke, VA, USA) were placed in the cannulae to maintain patency. All animals were given five days to recover from surgery and for the first three days after surgery, rats were treated daily with an analgesic agent (1 mg/kg Metacam, Boehringer Ingelheim, Ingelheim am Rhein, Germany) orally administered in drinking water.

## Intra-striatal infusions

The influence of aDLS DA receptor blockade on adjunctive drinking behavior was tested at early and late stage of training for SIPw and SIPa, namely during the acquisition of SIPw (Experiment 1, SIPw session 5 onwards, **Test 1 Effect of α-flu on Early SIPw**) and SIPa (Experiment 2, SIPa session 3 onwards, **Test 3, Effect of α-flu on Early SIPa**) and well-established SIPw (Experiment 2, SIPw session 20 onwards, **Test 2, Effect of α-flu on Late SIPw**) and SIPa (SIPa session 20 onwards, **Test 4, Effect of α-flu on Late SIPa**).

Before being tested, rats were habituated to the intra-aDLS insertion of the injector. Each test was preceded by intra-aDLS infusions (0.5 μl/side) of α-flupentixol (0, 6, 12 μg/side, made via 28-gauge steel injectors (Plastics One, Roanoke, VA, USA) lowered to the injection sites 2 mm ventral to the end of the guide cannulae. Infusions were made over 90 s using a syringe pump (Harvard Apparatus) and were followed by a 60 s period to allow diffusion of the infused drug or vehicle before injectors were removed and obturators were replaced. Test sessions began 5 min later. The effect of aDLS DA receptor blockade on adjunctive drinking was tested on rats from experiment 1 (**Test 1**) in a between subject design, and in rats from experiment 2 (**Tests 2, 3 and 4**) in a counterbalanced order following a Latin-Square design. Each infusion-day was followed by two baseline sessions.

## Histology

At the end of the experiment, rats were euthanized with an overdose of sodium pentobarbital (300 mg; Dolethal; Vétoquinol UK Ltd, Buckingham, UK) and then perfused transcardially with isotonic saline followed by 10% neutral buffered formalin. Brains were extracted and transferred to a 30% sucrose solution in 0.01 M PBS for 48 hours before being processed into 60 μm coronal sections using a Leica CM3050 S Research Cryostat. Sections were mounted and stained with Cresyl Violet.

Cannulae placements in the aDLS were verified using a light microscope by an experimenter blind to the behavioral results.

## Elevated Plus Maze (EPM)

The EPM tests were conducted during the dark phase of the dark/light cycle between 8am and 11am as previously described (3, 4), and immediately after a SIPw or SIPa session. The EPM (Viewpoint, France was situated at the centre of a dedicated room fitted with a camera attached to the ceiling right above it. The maze was elevated 50cm above the floor and comprised two open arms (45x10cm), two closed arms (45x10x45cm) and a central platform (10x10cm). The luminosity was measured in each portion of the maze and the light adjusted to reach an intensity of 40 Lux in the central platform, 50 Lux in the open arms and 30 Lux in the closed arms (13).

At the beginning of a test session, rats were placed on the central platform with their head pointing towards an open arm (always the same arm for all individuals). They were then allowed to explore the maze for 5 minutes while their movements were recorded. The colour of the EPM floor, the orientation of the maze in, and the visual cues placed on the walls of, the testing room were changed between each of the three tests to prevent any habituation.

Entries into, and time spent in, each open arm, were recorded as a measure of anxiety-related behavior. An anxiety score was calculated for each subject as a percentage of time spent in the open arms over the time spent in open and closed arms.

## Data and Statistical analyses

Group sizes were determined *a priori* by power analyses simulations (G*Power 3) (14) for one-way or Repeated-measures (RM) analyses of variance (ANOVA) and correlations based on published effect sizes of behavioural differences between alcohol and water coper rats on the one hand (4) and individual differences in the reliance on aDLS DA-dependent mechanisms in alcohol seeking (10) on the other hand.

Data presented as means ± SEM, individual data points or box plots [medians ± 25% (percentiles) and Min/Max as whiskers] were analyzed with STATISTICA-10 Software (Statsoft, Inc., Tulsa, OK, USA) or Statistical Package for Social Sciences (IBM SPSS, v 26, USA). Assumption for parametric analyses, namely homogeneity of variance, sphericity and normality of distribution were verified prior to each analysis with Cochran, Mauchly and Shapiro-Wilk’s tests, respectively. Where normality was substantially violated, data were Log transformed and where sphericity was violated, Greenhouse-Geisser correction was applied.

Behavioral data on acquisition or maintenance of SIPw or SIPa were analyzed using RM-ANOVAs with either time as within-subject factor alone or time or fluid as within-subject factor and group (HD vs LD or cluster) as between-subject factor.

The effect of aDLS DA receptor blockade on adjunctive drinking was analyzed with one-way- (**Test 1**) or RM-ANOVA (**Test 2, 3 and 4**) with treatment as between (**Test 1**) or within-subject factor (**Tests 2-4**) and group as between-subject factor (**Tests 2-4**).

Two-step K-mean cluster analysis (5, 10) was performed to identify groups of individuals whose reliance of adjunctive drinking on aDLS DA differed across tests. Thus, fluid intake under α-flupentixol treatment at Test 2 and 4 was averaged across doses and was expressed as percentage change from baseline (i.e., vehicle-treated rats); three subpopulations of rats were identified: Cluster 1 represented aDLS reliant water coper (WC) rats (n=11), Cluster 2 encompassed the marginally aDLS reliant WC rats (n=19) and Cluster 3 the aDLS reliant alcohol coper (AC) rats (n=8).

We additionally computed an index of differential reliance on aDLS DA of compulsive drinking of alcohol vs water, using the following equation: ⎜(sensitivity to aDLS at late SIPa - sensitivity to aDLS at late SIPw)/sensitivity to aDLS at late SIPw|. This enabled the analysis of whether the emerging reliance on aDLS control predicts individual tendency to rely on alcohol to cope with distress and the ensuing development of compulsive alcohol drinking (4).

Rats were also ranked according to their fluid intake (mL) before receiving α-flupentixol infusions (i.e., after the last three session of SIPw or SIPa) to identify individuals that increased their intake when alcohol was introduced in the SIP procedure.

The confirmation of significant main effects and differences among individual means were further analyzed using the Newman-Keuls post-hoc test, Dunnett's test (when comparing multiple time points to a single baseline) or planned comparisons, as appropriate. Significance was set at α ≤ .05 and effect sizes are reported as partial eta squared (η_p_^2^).

# References

1. Ansquer S, Belin-Rauscent A, Dugast E, Duran T, Benatru I, Mar AC, et al. (2014): Atomoxetine decreases vulnerability to develop compulsivity in high impulsive rats. *Biol Psychiatry*. 75:825-832.

2. Lopez-Grancha M, Lopez-Crespo G, Sanchez-Amate MC, Flores P (2008): Individual differences in schedule-induced polydipsia and the role of gabaergic and dopaminergic systems. *Psychopharmacology (Berl)*. 197:487-498.

3. Belin-Rauscent A, Daniel ML, Puaud M, Jupp B, Sawiak S, Howett D, et al. (2016): From impulses to maladaptive actions: the insula is a neurobiological gate for the development of compulsive behavior. *Mol Psychiatry*. 21:491-499.

4. Fouyssac M, Puaud M, Ducret E, Marti-Prats L, Vanhille N, Ansquer S, et al. (2021): Environment-dependent behavioral traits and experiential factors shape addiction vulnerability. *Eur J Neurosci*. 53:1794-1808.

5. Marti-Prats L, Belin-Rauscent A, Fouyssac M, Puaud M, Cocker PJ, Everitt BJ, et al. (2021): Baclofen decreases compulsive alcohol drinking in rats characterized by reduced levels of GAT-3 in the central amygdala. *Addict Biol*.e13011.

6. Pellon R, Ruiz A, Moreno M, Claro F, Ambrosio E, Flores P (2011): Individual differences in schedule-induced polydipsia: neuroanatomical dopamine divergences. *Behav Brain Res*. 217:195-201.

7. Moreno M, Gutierrez-Ferre VE, Ruedas L, Campa L, Sunol C, Flores P (2012): Poor inhibitory control and neurochemical differences in high compulsive drinker rats selected by schedule-induced polydipsia. *Psychopharmacology (Berl)*. 219:661-672.

8. Fouyssac M, Pena-Oliver Y, Puaud M, Lim NTY, Giuliano C, Everitt BJ, et al. (2022): Negative Urgency Exacerbates Relapse to Cocaine Seeking After Abstinence. *Biol Psychiatry*. 91:1051-1060.

9. Hodebourg R, Murray JE, Fouyssac M, Puaud M, Everitt BJ, Belin D (2019): Heroin seeking becomes dependent on dorsal striatal dopaminergic mechanisms and can be decreased by N-acetylcysteine. *Eur J Neurosci*. 50:2036-2044.

10. Giuliano C, Belin D, Everitt BJ (2019): Compulsive Alcohol Seeking Results from a Failure to Disengage Dorsolateral Striatal Control over Behavior. *J Neurosci*. 39:1744-1754.

11. Murray JE, Belin-Rauscent A, Simon M, Giuliano C, Benoit-Marand M, Everitt BJ, et al. (2015): Basolateral and central amygdala differentially recruit and maintain dorsolateral striatum-dependent cocaine-seeking habits. *Nat Commun*. 6:10088.

12. Paxinos G, Watson C (2013): *The rat brain in stereotaxic coordinates, 7th ed*. 7th ed. San Diego: Elesvier Academic Press.

13. Dilleen R, Pelloux Y, Mar A, Molander A, Robbins T, Everitt B, et al. (2012): High anxiety is a predisposing endophenotype for loss of control over cocaine, but not heroin, self-administration in rats. *Psychopharmacology (Berl)*.89-89.

14. Faul F, Erdfelder E, Lang AG, Buchner A (2007): G*Power 3: a flexible statistical power analysis program for the social, behavioral, and biomedical sciences. *Behav Res Methods*. 39:175-191.
